# Ecological Art Experience: How We Can Gain Experimental Control While Preserving Ecologically Valid Settings and Contexts

**DOI:** 10.3389/fpsyg.2020.00800

**Published:** 2020-05-15

**Authors:** Claus-Christian Carbon

**Affiliations:** ^1^Department of General Psychology and Methodology, University of Bamberg, Bamberg, Germany; ^2^Forschungsgruppe EPAEG (Ergonomics, Psychological AEsthetics, Gestalt), Bamberg, Germany; ^3^Bamberg Graduate School of Affective and Cognitive Sciences (BaGrACS), Bamberg, Germany

**Keywords:** empirical aesthetics, ecologically valid testing, art and perception, art experience, museum, gallery, real world

## Abstract

One point that definitions of art experience disagree about is whether this kind of experience is qualitatively different from experiences relating to ordinary objects and everyday contexts. Here, we follow an ecological approach that assumes art experience has its own specific quality, which is, not least, determined by typical contexts of art presentation. Practically, we systematically observe typical phenomena of experiencing art in ecologically valid or real-world settings such as museum contexts. Based on evidence gained in this manner, we emulate and implement essential properties of ecological contexts (e.g., free choice of viewing distance and time, large scale of artworks, and exhibition-like context) in controlled laboratory experiments. We found, for instance, that for large-scale paintings by Pollock and Rothko, preferred viewing distances as well as distances inducing the most intense art experiences – including Aesthetic Aha insights – were much larger than typical viewing distances realized in laboratory studies. Following Carbon’s (2019) terminology of measurement strategies of art experience, the combined use of “Path #1” (real-world context) and “Path #2” (mildly controlled, still ecologically valid settings and contexts) enables us to understand and investigate much closer what is really happening when people experience art.

## Introduction

Nearly all empirical research on aesthetics and most research on art experience are conducted in the context of experimental laboratories. Indeed, laboratory settings provide ideal conditions for setting up experiments in a standardized and rigorous way: they do, however, also create a specific context that is far from any typical context people encounter when they experience art in everyday life; for example, museums, art galleries, art happenings, or installations (Carbon, 2019). For an overview on the general problems of experimental, especially neuroexperimental, research about art experience, I would like to refer to a recent position article by Kubovy (2020).

The essential differences between typical experimental laboratory and ecological settings are manifold. In a very general sense, experimental laboratory settings might provide perfect systematic experimental conditions, but lack “ecological validity” (Brunswik, 1956) – see also “psychological ecology” (Lewin, 1943, p. 306) and “Lebensnähe” (English: “being close to real life,” see Lewin, 1927, p. 419). This lack of ecological validity comprises potentially reduced involvement (Deniaud et al., 2015) and decreased emotional processing (Schmuckler, 2001) – in the last consequence, we might even witness a lack or even loss of meaningfulness (Neisser, 1976). Specifically, in the field of art experience, it is quite evident that there are substantial differences in approaching, perceiving, and processing artworks in ecological contexts versus laboratory contexts. First, laboratory research is most often restricted to presenting copies of artworks, but not original artworks – simply because original artworks are not available for laboratory research because of their immense price and the fact that they would have to be transported out of the secured museum or stacks (Wolz and Carbon, 2014). Whether the originality factor has a great impact is still under debate. Results are quite mixed (Locher et al., 1999). One research group claims that their studies do not show an essential difference between the experience of art in the museum versus the laboratory (Brieber et al., 2015a), but they also present results that indicate influences on valuation and memory (Brieber et al., 2015b). Further research showed that the quality of experience is clearly changed with context (Wolz and Carbon, 2014; Pelowski et al., 2017). One reason for differences in the experience of art in different contexts could be related to the sample rather than the context itself: Often, museum visitors recruit from specific social groups (Hanquinet, 2013) that are not typically represented by participants attending a laboratory experiment, so studies researching context effects using a between-participants design might be biased in this respect (see Muth et al., 2017).

It should be noted that in specific cases the experience of art and art reproductions could be even better, richer, and deeper in a non-museum context. As was recently shown in a survey study by Bertamini and Blakemore (2019), high-quality reproductions allow a direct and close inspection of the artworks that is not possible in many art museums due to security issues. Additional promising results stem from preliminary studies in the domain of virtual reality testing (Janković et al., 2019). Importantly, the positive aspects of using copies cannot be emulated by presenting minimized versions of artworks on ordinary computer screens – we need, it seems, life-sized pictures printed in high quality.

A series of articles addressed the parameters of size, quality, and originality. Most of them actually concluded that they all influence the experience of art. Reproductions in original size are, for instance, considered more interesting, surprising, and pleasant by the participants (Locher et al., 2001), but they are also interpreted as being less complex (Locher et al., 1999). Large-scale (original) pictures additionally provoke a specific eye scanning behavior marked by a pronounced concentration on the central areas of the picture (Locher et al., 2008), and image size seems to modulate the observer’s viewing distance (Carbon, 2017) – at least in settings where this is possible, that is, mainly in real art-museum contexts.

Quality of depiction is still a rarely investigated topic in the field of art experience, but most originals provide a three-dimensional (3D) quality with canvas texture, protruding colors, and distinctive brush strokes (Carbon, 2016). This additional quality is mostly lacking in reductionistic laboratory research using common computer screens (see Locher et al., 2010), which narrows the overall experience to mostly plain visual stimulation and less pleasurable perception (Norman, 2002). Regarding originality, researchers have often found indications for a higher appreciation of original artworks: Viewers particularly appreciate the uniqueness of such works (Wolz and Carbon, 2014), and they are often well aware of the status of the artist who personally touched and created it (Newman and Bloom, 2012).

When directly observing museum visitors, it is quite evident that their viewing and inspection behavior is very different to what is found in typical laboratory contexts. First, most inspection in the laboratory is rather passive, but in the museum is typically active and explorative. We also freely choose the time we spend and the distance we take in to inspect pieces of art in the museum. These parameters and our specific pattern of approaching artworks are thus substantially, and probably qualitatively, different from the laboratory in their environmental setting. In a now classical study, Smith and Smith (2001) systematically investigated the visitors’ behavior while attending six masterpieces from the collection of the Metropolitan Museum of Art. The mean time visitors spent on viewing was 27.2 s. Subsequent studies with a similar methodological approach confirmed such long viewing times, for example, 28.6 s for viewing pieces from the permanent collection of The Art Institute of Chicago (Smith et al., 2017) and 32.9 s for viewing pieces from a temporary exhibition of Gerhard Richter’s work at the Neues Museum Nürnberg (Carbon, 2017). A study by Tröndle and Tschacher (2012), which analyzed visitors’ movement behavior covering much larger parts of a museum (Kunstmuseum St. Gallen) and much more diverse pieces of art, revealed much shorter average viewing times of about 10 s; some artworks, however, yielded viewing times similar to those found in the other studies (e.g., 34.5 s for the work “Antibild” by Günther Uecker made in 1974). Viewing times certainly depend on several factors such as the size of the observed picture (with larger pictures being viewed longer, see Carbon, 2017) or the social setting of where the artworks are attended (with people in a group looking at art longer than as an individual, see Carbon, 2017; Smith et al., 2017). Furthermore, viewing time is also modulated by reading or not reading the appending label (with visitors who read the label attending the artwork much longer – but only because they read the labels – and so effectively for a shorter period regarding the observation of the artwork as such, see Smith et al., 2017), or by the sheer number of artworks in an art show to be visited (Brieber et al., 2014).

Overall, there is overwhelming evidence that the context and the way of presenting artworks make a difference, especially regarding the richness of experience, the memory traces that are made, and the pleasure that is gained. When people are observed in the original habitat of experiencing artworks, for example, art galleries, museums, or special art shows, they behave qualitatively differently than in laboratory contexts. For instance, in a museum, they optimize their observation space, mostly taking in a much larger viewing distance and also using different viewing distances while constantly watching the artwork. Typical museum visitors also use far more time to inspect an artwork, and they return to many artworks after having fleetingly visited them before (Carbon, 2017). According to Brunswik (1956), we will not get “fully representative” (p. 67) research with laboratory-oriented research that ignores such typical viewing and inspection behavior, but we probably have at least a chance to go for “close-to-life systematic research” (p. 67) if we implement essential conditions by more ecologically valid study designs.

## The Present Study

In the present work, we consequently emulate and implement essential properties of the ecological-valid contexts of art perception (e.g., large scale, variable viewing distance, unrestricted inspection time, exhibition flair) within an experimentally controlled procedure. We thus follow the Path #2 approach proposed by Carbon (2019). We emulated a typical art gallery context by showing large-scale, high-quality reproductions printed on linen-like canvases, enabling large degrees of freedom of viewing with a very wide range of viewing distances. Participants approached the pictures one after another. During the inspection, no other person was attending the scene except the experimenter standing in the background. This should provide the ideal setup for the participant to fully concentrate on the artworks with no time limit and no time pressure. To gain a rich picture of their art experience, we employed this experience as a multidimensional construct as suggested by Faerber et al. (2010) for aesthetic appreciation. For the present study on abstract art, we employed the following variables: (1) *liking* (German: Gefallen), (2) *power(fulness)* (Kraft), (3) *interesting(ness)* (interessant), (4) *emotional (value)* (emotional), and (5) *3D impression* (3D Wirkung); finally, we asked whether an Aesthetic Aha insight moment occurred while viewing the artwork. *Liking* assesses how personally pleasing an artwork is for the participant; this variable is employed in most aesthetic studies addressing preferences (Faerber and Carbon, 2012; for an overview of operationalization of this variable, see Faerber et al., 2010; Marin et al., 2016; e.g., Muth and Carbon, 2013). *Powerfulness* (Pepperell, 2011) was employed to reflect the other classical axis of preference besides pleasantness (Ortlieb et al., 2016), which is often discussed in aesthetic theories such as those of Edmund Burke or Immanuel Kant to describe how impressive an artwork is. *Interestingness* represents the important component of aesthetic experience, which triggers the motivation for a deep inspection (Silvia, 2005b,c). However, this variable is often neglected or suppressed in aesthetic research because of biased reliance on aspects of beauty or pleasantness (Turner and Silvia, 2006; Silvia, 2008; Muth et al., 2015a). *Emotional (value)* represents the personal assessment of how emotional the impression of an artwork was and corresponds to the facet *valence* from the aesthetic appreciation concept of Faerber et al. (2010). It primarily reflects the affective response of a person. *3D impression* was specifically employed for the abstract expressionist paintings by Marc Rothko and Jackson Pollock used in the present exploratory study. Works by these artists are often described as triggering experiences of visual depth that immerse the viewer (Emmerling, 2003). Last but not least, *Aesthetic Aha* represents a sudden insight into perceptual Gestalt (Muth and Carbon, 2013). The Aesthetic Aha (as a concept) is a part of a typical experience of artistic epiphany (Carbon, 2019), which typically leads to an increased liking of the artifact from which the person had the insight (Muth and Carbon, 2013) and can even lead to transformative effects (Pelowski, 2015). Just as an example: When we enter the titular church of San Pietro in Vincoli, Rome, Michelangelo Buonarroti’s *Moses* – sculpted 1513–1515 of the finest Carrara marble – makes a clear impression on us by its sheer size of more than 2 m in height. But only by close inspection do we become aware of the lively and energetic character of Moses. After some time, many visitors recognize female body shapes in the swirling beard – they perceive gestalts instead of background information. Strong experiences such as having an Aesthetic Aha are particularly interesting to investigate in ecologically valid contexts because the deeper effects of aesthetic experience are quite rare in standard laboratory contexts, and therefore their existence might even be questionable if we always carry out our aesthetic research in laboratory settings that are mostly far from reality.

## Experiment

### Methods

#### Participants

We tested 10 participants (eight female, *M*_*age*_ = 26.1 years) who had no special training in the arts, but were mostly interested in art (*M* = 5.1 on an eight-point scale from 0 = no interest at all to 7 = very high interest). As the study used paintings by Mark Rothko and Jackson Pollock as material, we specifically asked for the participants’ knowledge of both artists via two separate eight-point scales ranging from 0 = no knowledge at all to 7 = very high. Most of the participants had no particular knowledge of Rothko (only one participant indicated knowledge greater than 4 on this scale), whereas Pollock was somewhat more well-known (three participants indicated knowledge about Pollock as greater than 4 on the respective scale). Participants were invited to join a “small art show organized by the department” – we did not provide explicit knowledge, neither on the artists involved nor any other information about the paintings. The participants were recruited from several lectures. They were mainly psychology students, and they received course credit for their participation. All participants had normal or corrected-to-normal vision, as tested by the Snellen Eye chart test. Normal color vision was shown by all participants through fully correct responses in a short self-fabricated version of the Ishihara Color test.

#### Stimuli

Six large-scale abstract expressionist paintings (Table 1), three by Marc Rothko (an American painter of Jewish–Litvak descent who lived from 1903–1970, mostly known for his abstract expressionist paintings) and three by Jackson Pollock (an American painter who lived from 1912 to 1956 and was a major figure in the abstract expressionist movement) were used as stimulus material.

**TABLE 1:** List of artworks used in the present study, printed on linen-like canvases and mounted on wooden stretcher frames.

**Artist**	**Title**	**Year**	**Original and used size**
Mark Rothko	No. 7 (Dark Brown, Gray, and Orange Brown)	1963	162.5 × 175.5 cm [106.8 × 117.0 cm]
	No. 21 (Red, Brown, Black, and Orange)	1951	162.5 × 241.5 cm [106.8 × 162.0 cm]
	Untitled (Yellow and Blue)	1954	186.7 × 242.9 cm [106.8 × 140.0 cm]
Jackson Pollock	Number 1A	1948	264.2 × 172.7 cm [167.0 × 106.8 cm]
	Blue Poles (Number 11)	1952	488.9 × 212.1 cm [242.0 × 106.8 cm]
	Full Fathom Five	1947	76.5 × 129.2 cm [106.8 × 189.5 cm]

*Sizes are shown as width × height. Realized sizes in the experiment are indicated within brackets.*

### Procedure

The study comprised two major experimental blocks. Both blocks were characterized by six sub-blocks devoted to one artwork each. The order of artworks was randomized for each person and was fixed across blocks. All instructions were given in German. In both blocks, participants had to evaluate their experience of each artwork on a series of five seven-point Likert scales (1 = very weak, 7 = very strong) representing different dimensions of aesthetic experience: (1) liking (German: Gefallen), 2) power(fulness) (Kraft), (3) interesting(ness) (interessant), (4) emotional (value) (emotional) and (5) 3D impression (3D Wirkung). Additionally, we asked the participants whether they had experienced an Aesthetic Aha insight (Muth and Carbon, 2013) while viewing the artworks – if so, they were requested to describe the aha experience in their own words. In the first block (“assigned distances” condition), the experimenter situated the participants at various predefined distances in front of the paintings (the experimenter guided them to the respective subtly marked positions on the floor by hand; they were instructed to inspect the painting from these positions and evaluate it according to the questions provided while staying there) – the empirical distances were also registered and measured exactly later on, as participants tend not to fully fix their positions to those assigned. The order of the eight predefined distances was randomized for each sub-block and for each participant. The range of distances was from very near to far, that is, 0.5, 1.0, 1.5, 2.0, 3.0, 4.0, and 5.0 m, which are typical distances that can be observed in real museum contexts (Carbon, 2017), plus a very far distance of 10.0 m. In the second block (“self-chosen distance” condition), after having been massively familiarized with the presented artworks, participants were asked to make their own, preferred choice of distance to finally view each painting in an optimal way. For all trials, we let participants view the respective artwork as long as they wanted; there was no time pressure and no time limit, so that participants had the opportunity to deeply process each artwork. In order to assist this deep processing and to reduce any distraction, the participants’ assessments were verbally requested and were then written down by the experimenter.

Prior to the experimental session, written informed consent was obtained from each participant. Additionally, we conducted a personality test concerning the Big Five, based on 21 items [Big Five Inventory short version (BFI-K), Rammstedt and John, 2005]. After the experiment, participants were fully informed about the background of the study and allowed to ask questions. Persons who did not consent were not included in the study – but this did not happen in the course of the study. All data were collected anonymously. The entire procedure took 2–3 h per person.

### Results and Discussion

We were mainly interested in gaining insight into three aspects: (1) how viewing distance changes the aesthetic experience of large-scale artworks, (2) how Aesthetic Aha insights modulate the aesthetic experience, and (3) how liking of an artwork can best be predicted by other qualities of aesthetic experience. The data were processed by RStudio 1.2.5001 with *R* 3.6.1, using the *R* toolbox *psych* for calculating effect sizes. Linear mixed-effects analysis was conducted via toolbox *lme4* (Bates et al., 2015).

### Data Basis

We registered no loss of data for any of the participants yielding 324 data points per person, that is, 5 + 1 [Aha] = 6 data points per picture and distance in blocks yielding 6 × 8 × 6 = 288 data points for Block 1, and 5 + 1 [Aha] = 6 data points per picture yielding 36 further data points for Block 2. All in all we obtained 324 data points per person, so 3,240 overall.

### Strategy of Analyzing the Data

The analysis of data will start with the mean data from Block 1, where we let the artworks be experienced at specifically assigned viewing distances. After that, we will focus on the data of Block 2 where we let the participants find the optimal viewing distance for each artwork. The mean data of both blocks will be compared via linear mixed effects to reveal any benefit of the mode of how viewing distance is established – fixedly assigned (Block 1) versus self-chosen (Block 2). We then explicitly analyze the relationship between assigned distance and the quality of art experience. We will do this by looking at all artworks differently as we believe that certain artworks ask for specific viewing distances; for instance, larger-scale pictures often implicitly need greater distances to fully appreciate them (Carbon, 2017). All these analyses include the five focus variables of aesthetic experience (3D impression, emotional, interesting, liking, and power) plus the quality of whether an Aesthetic Aha effect takes place when inspecting the artwork. In order to find out whether viewing distance is more a general factor or a viewer-specific one, we will furthermore test viewing distance within linear mixed models as fixed versus random slopes. As liking is a central variable in art experience, we will then focus on this specific target variable when looking at the impact of viewing distance. For the self-chosen distances, we will also look at the histogram of viewing distances to get an impression of how single viewers differ in their idiosyncratic interpretation of an optimal distance for specific artworks. Lastly, we will analyze the trials in which an Aesthetic Aha happened in comparison with trials where such Aesthetic Ahas were not available – here we were especially interested in the impact on the other five variables capturing the concept of art experience.

### Overview of Aesthetic Experience Data Including Aesthetic Aha

**FIGURE 1:**
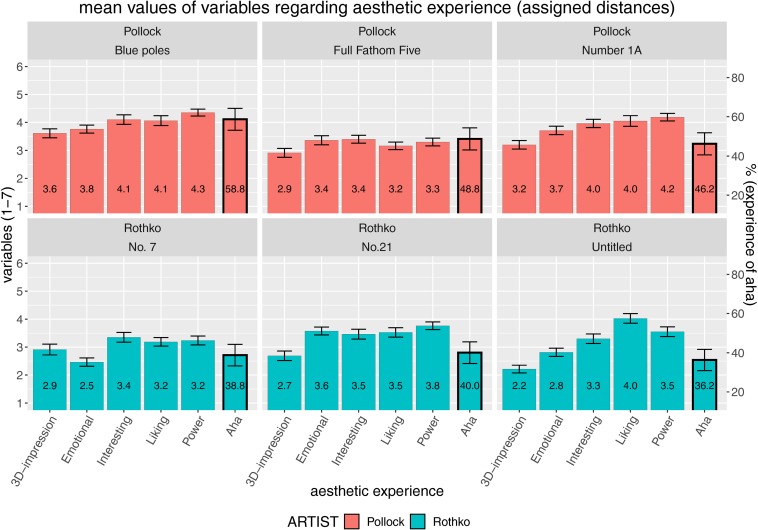
Evaluations with assigned distances: mean values for aesthetic experience comprising five different quantitative variables (3D impression, emotional value, interestingness, liking, and powerfulness) plus the percentage of having experienced an Aesthetic Aha insight, all split according to artist (paintings by Pollock in the top row and paintings by Rothko in the bottom row). Error bars indicate standard error of the mean (SEM).

For an initial inspection of the data, we examined the mean values of aesthetic experience and the mean percentage of having experienced an Aesthetic Aha insight (Muth and Carbon, 2013). First, we analyzed the mean data of the first experimental block where fixed distances were assigned to the participants (Figure 1). From the mere visual inspection of these mean values (averaged across distances), it is clear that the aesthetic profiles differed among the artworks and that Pollock paintings in particular generated Aesthetic Aha insights quite often, on average, in approximately half of all inspections within a range of 46.2–58.8% of all cases.

**FIGURE 2:**
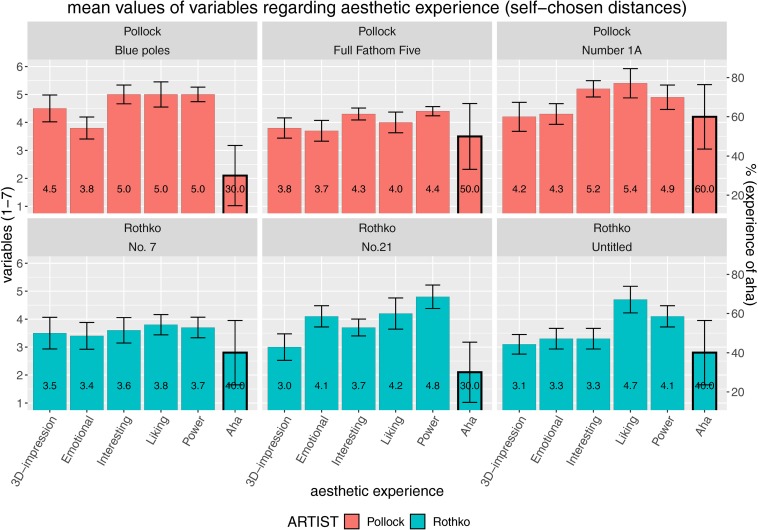
Evaluations with self-chosen distances: mean values for aesthetic experience comprising five different quantitative variables (3D impression, emotional value, interestingness, liking, and powerfulness) plus the percentage of having experienced an Aesthetic Aha insight, all split according to artist (paintings by Pollock in the top row and paintings by Rothko in the bottom row). Error bars indicate standard error of the mean (SEM).

Second, we analyzed the mean data of the second experimental block where the distances were self-chosen by the participants (Figure 2). The data were similar – but obviously, aesthetic experience was at a higher level in general when participants were allowed to choose the viewing distance on their own. Note: We have to be cautious in interpreting this higher level as a direct outcome of the assigned distance versus self-chosen distance condition, because the self-chosen condition was always executed after the assigned distance condition. Thus, this effect can also be explained by a deeper elaboration as such. It is, nevertheless, important to stress that this effect was probably not caused by mere exposure (Zajonc, 1968) as we did not find an effect of trial number in the assigned distance condition on liking [linear mixed Model #0 from Table 2 tested against the same model with additional trial number as fixed slope, *p*(χ^2^(7)) = 0.2268, not statistically significant (n.s.)] – thus, the mere frequency of having inspected an artwork did not significantly yield higher aesthetic appreciation.

**TABLE 2:** Linear mixed-effects analysis of different models in comparison to a simple base model (Model #0).

**Dependent variable/tested model**	**df**	**AIC**	**logLik**	***R*^2^**	***p*(*χ*^2^)**
3*D impression*					
#0: *Base* (random intercepts)	4	1,783	−887	0.187	
**#1: *+* FS distance**	**11**	**1,764**	−**871**	**0.238**	**<0.0001**
#2: *+* RS distance (by artists)	13	1,766	−870	0.241	0.4051, n.s.
*Emotional* value					
**#0: *Base* (random intercepts)**	**4**	**1,664**	−**828**	**0.221**	
#1: *+* FS distance	11	1,671	−824	0.232	0.4113, n.s.
#2: *+* RS distance (b*y artist*s)	13	1,675	−824	0.232	0.8516, n.s.
*Interestingness*					
#0: *Base* (random intercepts)	4	1,705	−848	0.235	
**#1: *+* FS distance**	**11**	**1,703**	−**840**	**0.258**	**0.0264**
#2: *+* RS distance (b*y artist*s)	13	1,706	−840	−259	0.7332, n.s.
*Liking*					
#0: *Base* (random intercepts)	4	1,790	−891	0.173	
**#1: *+* FS distance**	**11**	**1,759**	−**869**	**0.242**	**< 0.0001**
#2: *+* RS distance (b*y artist*s)	13	1,763	−869	0.242	0.9604, n.s.
*Powerfulness*					
#0: *Base* (random intercepts)	4	1,638	−815	0.207	
**#1: *+* FS distance**	**11**	**1,632**	−**805**	**0.238**	**0.0045**
#2: *+* RS distance (by artists)	13	1,634	−804	0.240	0.5418 n.s.

*For each dependent variable, the best-fitting model, while being parsimonious, is indicated by bold face. FS, fixed slopes (fixed factors); RS, random slopes (random factors); df, degrees of freedom; *R*^2^, coefficient of determination, based on the likelihood-ratio test; *p*(χ^2^), probability of accepting a significant effect despite a non-existent difference regarding the more complex versus the one-step less complex model.*

We tested the differences between both viewing conditions (assigned vs. self-chosen distances) by means of linear mixed-effects analyses as shown in Table 3. With the exception of Aesthetic Aha, all aesthetic experience variables showed higher values in the self-chosen viewing distance condition than in the assigned distance condition.

**TABLE 3:** Mean values of aesthetic experience variables for assigned (experimental Block 1) versus self-chosen (Block 2) distances.

**Variable**	***M* (assigned)**	***M* (self-chosen)**	***p***	**Cohen *d***
3D impression	2.92	3.68	<0.0001	0.370 “small to medium”
*Emotional*	3.28	3.77	0.0025	0.263 “small”
*Interestingness*	3.60	4.18	0.0005	0.303 “small to medium”
*Liking*	3.67	4.52	<0.0001	0.400 “small to medium”
*Powerfulness*	3.73	4.48	<0.0001	0.418 “small to medium”
*Aha*	0.448	0.417	0.6172, n.s.	–

*Statistical tests were conducted by means of linear mixed-effects analyses, employing models with random intercept effects for participants and artworks and the respective target variable as fixed slope effects. Effect sizes (expressed as Cohen *d*) are qualified according to the suggestions of Cohen (1988).*

This finding is particularly interesting as it shows that taking an own, optimally suiting viewing distance is quite important for the aesthetic experience of artworks. This is typically not acknowledged in laboratory research where distances are mostly fixed and even fixed at a very close distance.

### Aesthetic Experience in Relation to the Viewing Distances

**FIGURE 3:**
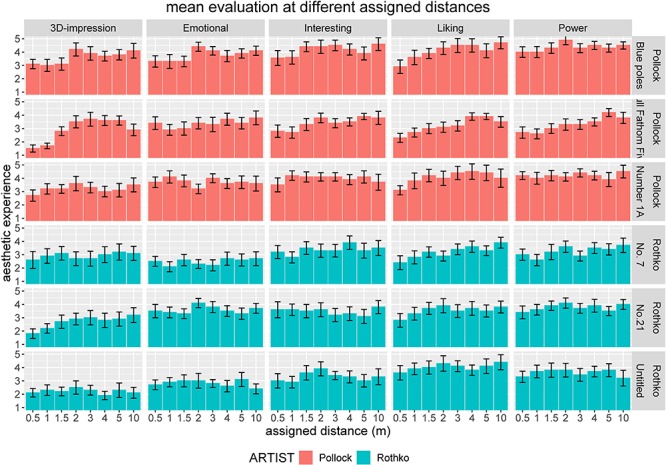
Impact of assigned viewing distance on aesthetic experience comprising five different quantitative variables (3D impression, emotional value, interestingness, liking, and powerfulness) split according to artworks (paintings by Pollock in the top rows and paintings by Rothko in the bottom rows). Error bars indicate standard error of the mean (SEM).

In the following section, we will focus on the multidimensional construct of aesthetic experience comprising five quantitative variables that participants assessed for each artwork and a qualitative variable indicating whether an Aesthetic Aha insight was experienced. An initial visual inspection of the data for the first experimental block with assigned distances (Figure 3) already indicated that the respective assigned distance had an influence on several variables of aesthetic experience.

We statistically tested the impact of assigned distances on aesthetic experience by employing separate series of linear mixed-effects analyses with the independent measure viewing for each of the five variables of the construct of aesthetic experience, that is, (1) 3D impression, (2) emotional value, (3) interestingness, (4) liking, and (5) powerfulness. As base model (Model #0), we defined only random intercepts for participants and artists. Then we successively increased the complexity of the model by first entering distance as fixed slopes – FS (fixed factors) – (Model #1) in order to test the impact of distance on aesthetic experience and then by adding random slopes for participants and artists (Model #2). Visual inspection of residual plots did not reveal any obvious deviations from homoscedasticity or normality. *P*-values were obtained by likelihood-ratio tests of the subsequent models against the base model. The coefficient of determination for each model was calculated via a likelihood-ratio test utilizing the toolbox *MuMIn* (Barton, 2019). See Table 3 for detailed results.

Linear mixed-effects analysis revealed that, with the exception of emotional value, all quantitative variables of aesthetic experience were impacted by assigned viewing distance. Furthermore, this impact was quite constant for both artists, as indicated by a non-significant information increase when adding random slopes for distances-by-artists.

**FIGURE 4:**
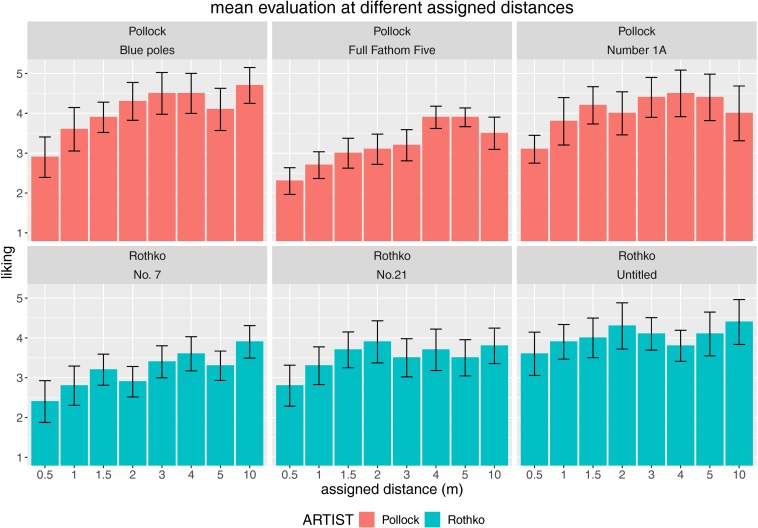
Impact of assigned viewing distance on liking for each employed artwork (paintings by Pollock in the top row and paintings by Rothko in the bottom row). Error bars indicate standard error of the mean (SEM).

We inspected this uniformity of effect in further detail by focusing on the aesthetic experience variable *liking*, which showed a particularly strong modulatory power of distance – the strongest in fact, as revealed by the linear mixed-model-effect analyses. We revealed a clear increase in liking the farther away the viewpoint of the participants was, with an optimal viewing distance regarding the modal value of approximately 3 to 4 m (Figure 4), which is substantially farther away than in the study by Carbon (2017) when observing the natural (on-site) viewing behavior in a Gerhard Richter art show – *M* = 1.72 m [1.49–2.12 m]. As the Richter paintings were a bit smaller than the stimuli employed in the present study, we applied the formula of empirical viewing distance in relation to the picture size provided by Carbon (2017). This yielded smaller predicted distances than observed ones, for example, 1.71 m for the smallest painting – Rothko’s “No.7 Dark Brown, Gray and Orange Brown” with a size of 1.25 m^2^, and 1.98 m for the largest painting – Pollock’s Blue Poles (Number 11) with a size of 2.58 m^2^. It seems that the optimal viewing distance is related not only to the canvas size but also to the subject or the specific artistic style, which differed in both studies.

**FIGURE 5:**
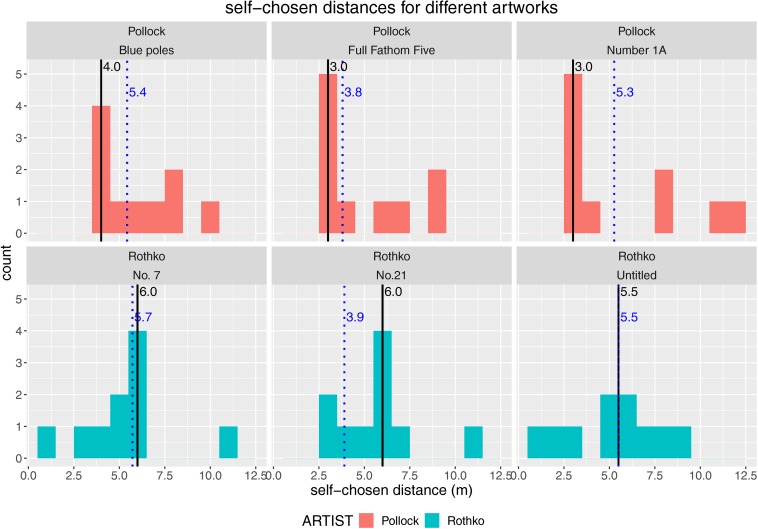
Histograms of self-chosen distances for each employed artwork (paintings by Pollock in the top row and paintings by Rothko in the bottom row). Black solid vertical lines plus black numbers show the modes of the distributions; blue dotted vertical lines plus blue numbers show the medians of the distributions.

With the second experimental block, we further elaborated the investigation of optimal viewing distances. Here, we explicitly asked and allowed participants to choose their optimal viewing distance to gain the strongest aesthetic experience of the artworks. We found that participants chose quite large distances to view the artworks optimally. Taking the most frequently chosen viewing distances into account, we revealed a range of [3.0–4.0 m] for Pollock paintings and [5.5–6.0 m] for Rothko paintings (Figure 5).

So again, empirical viewing distances in an ecologically valid context were much farther away than typical distances realized in typical experimental laboratory settings.

**FIGURE 6:**
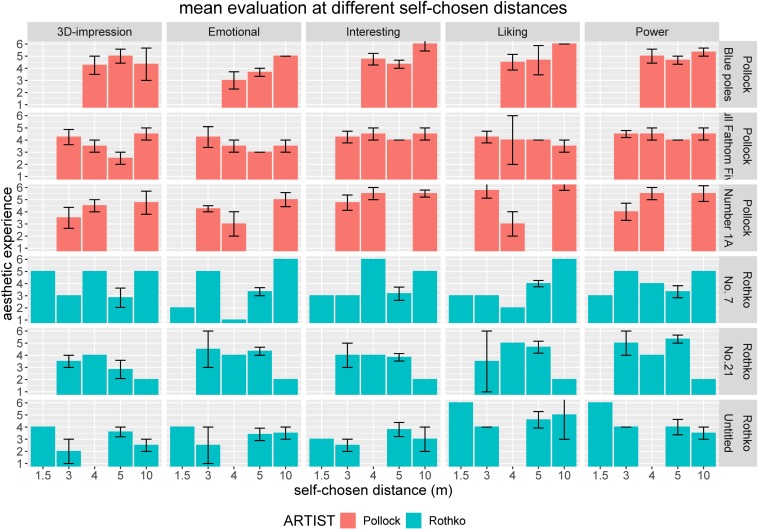
Self-chosen viewing distances relating to aesthetic experience comprising five different quantitative variables (3D impression, emotional value, interestingness, liking, and powerfulness) split according to artworks (paintings by Pollock in the top rows and paintings by Rothko in the bottom rows). Error bars indicate standard error of the mean (SEM).

Self-chosen viewing distances were also accompanied by different aesthetic experiences (Figure 6). For Pollock paintings particularly, we observed most of the higher quality aesthetic experiences at farther distances, whereas Rothko paintings were appreciated at medium and sometimes also at closer distances, which were still much farther away than typically realized in laboratory experiments.

### Qualities of an Aesthetic Aha

**FIGURE 7:**
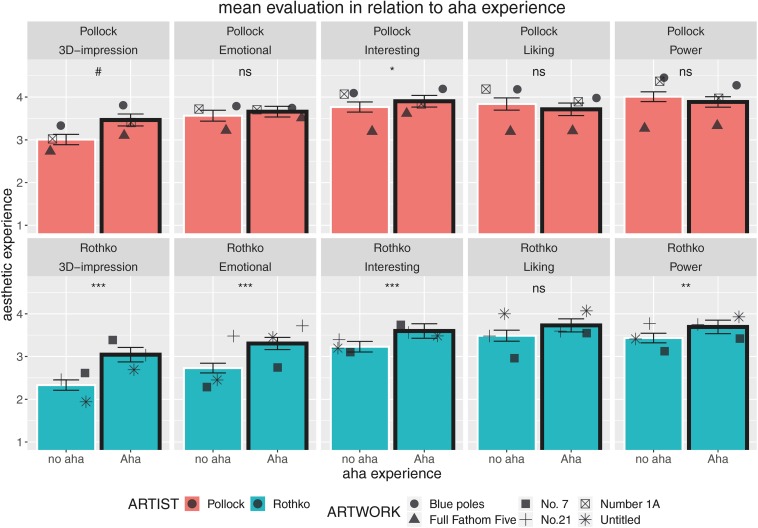
Experimental block on assigned distances: Relationship between having an Aesthetic Aha insight (Muth and Carbon, 2013) and aesthetic experience comprising five different quantitative variables (3D impression, emotional value, interestingness, liking, and powerfulness) split according to artist (paintings by Pollock in the top row and paintings by Rothko in the bottom row). Dots show the mean data for specific paintings. Error bars indicate standard error of the mean (SEM). Differences between “no aha” and “Aha” trials were tested for statistical significance via linear mixed-effects analysis with 2 × 5 single models with the only fixed factor representing an aha absent or present (ns, non significant, #*p* < 0.1, *p < 0.05, **p < 0.01, ***p < 0.001).

Based on previous research on the so-called Aesthetic Aha insight in which people report increased pleasure when having such an insight experience, for example, shown for the visual domain (Muth and Carbon, 2013; Muth et al., 2013) but also quite recently for haptics (Muth et al., 2019b), we analyzed the impact of experiencing an Aha on the here-targeted variables of aesthetic experience. Mean data for each of these variables shown in Figure 7 indicate a positive influence of experiencing an Aha insight on the aesthetic experience of artworks, especially for the paintings of Mark Rothko. For Rothko paintings, we revealed numerical benefits for all variables and significant increases for all variables except *liking* (Figure 7). For Pollock paintings, Aesthetic Aha showed only a significant increase for the variable *interesting*. These findings are particularly interesting as the Aesthetic Aha effect was mainly attributed to a benefit concerning pleasure, but was speculated to impact the full range of aesthetic experience as well. In the original study by Muth and Carbon (2013), pleasure was operationalized via the German term “Gefallen” and translated to “liking.” However, in English, it seems to be better captured by the term “pleasing”; in the context of haptics, for instance, we asked for “pleasingness” as well as “pleasantness,” see Muth et al. (2019b). The results of the present study, at least for the Rothko paintings, would partly support this view, but the Aha insight benefit might also be limited to certain kinds of aesthetic displays.

### Predicting Liking Through Other Dimensions of Aesthetic Experience

Finally, we were interested in how liking of an artwork can best be predicted by other dimensions of aesthetic experience. In order to test this, we employed linear mixed-effects analyses with increasingly complex models.

As indicated by Table 4, we identified as best fitting a model that took all aesthetic experience variables into account as fixed factors, plus the variables *interestingness* and *powerfulness* as random slopes by artworks as well as participants. For this “Model #2,” the fixed factors *interestingness* and *powerfulness* showed moderately large significant effects; both factors positively related to the *liking* of artworks.

**TABLE 4:** Linear mixed-effects analysis for models aiming to predict *liking* by the four aesthetic experience variables 3D impression, emotional value, interestingness, and powerfulness.

**Tested model**	**df**	**AIC**	**logLik**	***R*^2^**	***p*(*χ*^2^)**
Base *M*odel #0	8	1442	−713	0.589	
Model #1a	13	1433	−704	0.604	0.0021
Model #1b	13	1402	−688	0.628	<0.0001
**Model #2**	**18**	**1400**	−**682**	**0.637**	**0.0305**
**Model #2**	**Estimate**	***t***	**df**	***p***	**Cohen *d***
FE 3D impression	0.014	<1	388.5	0.7262, n.s.	–
FE emotional	0.075	1.50	314.5	0.1335, n.s.	–
FE interesting	0.425	4.23	6.4	0.0047	0.3818 “medium”
FE power	0.363	5.34	8.7	0.0005	0.4820 “medium”

*Base Model #0 contains only these four variables as fixed factors, plus participants and artworks as random intercepts. Model #1a and Model #1b add random slopes for *interestingness* and *powerfulness* by artworks and participants, respectively. Model #2 combines Models #1 and #1b by adding random slopes by artworks and participants. Best-fitting model, while being parsimonious, is indicated by bold face. FS, fixed slopes (fixed factors); df, degrees of freedom; *R*^2^, coefficient of determination, based on the likelihood-ratio test; *p*(χ^2^), probability of accepting a significant effect despite a non-existent difference regarding the more complex versus the one-step less complex model. For the best-fitting model, statistics about fixed effects are given in detail. Effect sizes (expressed as Cohen *d*) are qualified according to the suggestions of Cohen (1988).*

## General Discussion

The main aim of the present exploratory study was to add insights about ecologically valid behavior of art perceivers in a museum context. We created an experimental setting that emulates typical properties of such a context by organizing a small art show where people were allowed to view artworks without time constraints at different viewing distances. In a first experimental block, they were assigned to fixed distances, and then in the second experimental block, they were asked to freely choose the distances to optimally view the artworks on their own.

First, we observed an impact of viewing distance on aesthetic experience. Based on the specifics of the inspected painting, we revealed certain distances that were mostly much larger than are typically employed in laboratory-based research on aesthetics where the distance is mainly defined by the optimal viewer-screen distance. Here, with the help of large-scale prints of artworks, people had Aesthetic Aha insights quite often and benefited from self-chosen distances. As soon as they were able to choose their personal viewing distance, this indeed was a kind of optimal one in order to maximize the level of aesthetic experience. Such self-chosen distances were mostly in the range of 3 m up to 6 m, with smaller distances for Pollock than Rothko paintings. But even when participants were assigned fixed distances, they showed a specific pattern of viewing distance relationship with certain aesthetic experiences. Especially *liking* of a painting benefited from farther distances, but also the powerfulness, the 3D impression and the interestingness of paintings were influenced by the position from which the viewer inspected the artworks.

When participants reported an Aesthetic Aha insight, we also detected intensified aesthetic experience, specifically for Rothko paintings. They reported an increased 3D impression and more emotional value; they found the artworks more interesting and characterized them as being more powerful. Interestingly, such Aha insights did not trigger higher levels of liking, a key variable of aesthetic experience which Muth and Carbon (2013) proposed to be impacted by Aha insight moments (see Carbon, 2010). At the moment, we can only speculate as to why neither Rothko nor Pollock paintings were better liked when Aesthetic Aha happened, but probably the aesthetic experience, which is triggered by such Aha moments is much broader than was initially suggested. Thus, the term “Aesthetic Aha” also seems to be a suitable term for referring to a broad concept of processes being involved in epiphany moments of insight (see Carbon, 2019). Further research has to investigate the impact and reach of Aesthetic Aha effects, and especially the role of interestingness in this respect. Interestingness was strongly impacted by Aesthetic Aha for Rothko as well as Pollock paintings in the current study and is a variable of much interest in recent streams of empirical aesthetic studies (Silvia, 2005a, 2008; Muth et al., 2015a). And indeed, whereas classical studies and theories mainly refer to beauty and liking, more recent ones – especially those investigating contemporary or “challenging art” (Belke et al., 2015) – do focus on interest for the inspection (Silvia, 2005b) or focus on the promise to understand parts of an artwork (Muth et al., 2015a, 2019a; Muth and Carbon, 2016).

We also looked at the classical question of what dimensions of aesthetic experience predict the liking of a painting. Among our targeted variables, we again revealed not only interestingness, but also powerfulness, as promising candidates for predicting how much people will like a painting. Knowledge about the relationship between powerfulness and liking is still very limited, although initial research exists (Pepperell, 2011). Sometimes even different types of powerfulness are discussed, for example, perceptual versus cognitive aspects (Muth et al., 2015a), both being influenced by insights and by ambiguity – and both phenomena playing a crucial role in abstract art as utilized in the present study. Research on interestingness is much more developed in this respect because of some key publications on interest (Silvia, 2005b; Muth et al., 2015a) and interestingness (Silvia, 2005c; Faerber et al., 2010). It is quite clear that, similarly to powerfulness, interest is often not directly connected to liking, and probably even less connected to beauty aspects, especially in modern art where challenge, the promise of insight, and actual insight are much more important. These now more-focused concepts are very closely linked with interest as they are perfect triggers to attend and elaborate an artwork, and interest seems the key concept for such curious behavior (Silvia, 2008). The type of artwork and especially the meaningfulness of an artwork might modulate the relationship between liking and interest. Whereas world-renowned paintings create a natural interest at the same time as being liked, contemporary artworks might be primarily qualified as being interesting but not primarily liked. An again-different relationship can be observed for more kitschy art, which is often liked but does not trigger too much interest (Ortlieb and Carbon, 2019). The elaboration over time and inspection might also change the flexible relationship between interest and liking, with challenging art being liked only after deep elaboration (see Carbon and Leder, 2005) and less innovative art being devalued after sufficient elaboration (Belke et al., 2015) or after one has “solved” the message of a picture (Muth et al., 2015b). Only the joint effort of many research groups investigating the details and the moderators of such essential relationships among aesthetic concepts, which were and still are the cause of many endless debates in the field of empirical aesthetics, might uncover the real drivers for and the nature of aesthetic experience.

Finally, it is important to stress that effects found in the present study cannot be easily generalized to other artworks (for instance, to more figurative, more popular, more easy-to-process art), other settings, and other parameters. The study, however, illustrates how impactful certain variables – such as viewing distance, elaboration, and even whether viewing distances are prefixed or freely chosen – are with respect to experiencing art and triggering Aesthetic Aha moments. Particularly, the perfect viewing distance allowing for “optimum” aesthetic experience probably depends very much on the specific material inspected: Most large-scale pictures, like the ones used in the present study, ask for much larger viewing distances than some incredibly detailed small-sized pictures (e.g., pictures from the Dutch 16th-century naturalist miniature tradition), which develop their full aesthetic impact only when inspected very closely.

## Conclusion

If we aim to understand and investigate true art experience as a rich phenomenon of deep elaboration and strong affective and cognitive impact, we first need to trigger such experiences such as art epiphany (Carbon, 2019). We can gain knowledge about the typical factors triggering and supporting such experiences by analyzing the typical settings of art galleries and the behavior visitors show in them by employing observation studies in the field. On the basis of this knowledge, we can implement ecologically valid settings and employ the required measurement strategies recruited from the powerful toolbox of experimental and systematic empirical research. This strategy assists the aim of approaching closer to the real phenomenon of art experience without losing scientific control.

## Data Availability Statement

The datasets generated for this study are available on request to the corresponding author.

## Ethics Statement

The study was given ethical approval by the local ethics committee of the University of Bamberg (Bamberg, November 23, 2014, signed by the chairperson of the ethics committee). The patients/participants provided their written informed consent to participate in this study.

## Author Contributions

Idea, planning, procedure, analysis, report, writing, critical reflection, and literature review was done by C-CC.

## Conflict of Interest

The authors declare that the research was conducted in the absence of any commercial or financial relationships that could be construed as a potential conflict of interest.
